# The effect of health literacy and self-management efficacy on the health-related quality of life of hypertensive patients in a western rural area of China: a cross-sectional study

**DOI:** 10.1186/s12939-017-0551-9

**Published:** 2017-07-01

**Authors:** Chenli Wang, Juntao Lang, Lixia Xuan, Xuemei Li, Liang Zhang

**Affiliations:** 1 School of Medicine and Health Management, Tongji Medical College, Huazhong University of Science and Technology, Wuhan, Hubei 430030 China; 2 ZhongShan Hospital Fudan University, Shanghai, China; 3 Affiliated Hospital of Weifang Medical University, Weifang, Shandong China

**Keywords:** Effect, Health literacy, Self-management efficacy, HRQL, Hypertension

## Abstract

**Background:**

Hypertension is a common and frequently occurring chronic disease of the cardiovascular system. Besides the pathological factors, the occurrence and exacerbation of hypertension are also associated with many factors of lifestyle and behaviors. Thus hypertensive patients’ Health-related quality of life (HRQL) is not only influenced by the disease itself but also by many subjective factors such as health literacy and self-management efficacy, especially in the deeper part of southwestern China and thus is less developed compared to the other places. The purpose of this study was to examine the association between the HRQL of hypertensive patients and health literacy and self-management efficacy as well as how they affect the HRQL, so as to provide a theoretical reference for improving the HRQL of patients with hypertension in less developed areas.

**Methods:**

This was a cross-sectional study of baseline data from a clustered randomized controlled trial. The study design had passed a cross-national peer review and accepted grants by the China Medical Board. It was also registered in the Chinese Clinical Trial Registry (ChiCTR-OOR-14005563).

A standardized questionnaire adapted from a previous validated WHO questionnaire was used for the survey which included detailed questions about patient’s socio-demographic characteristics and self-reported information.

Patients’ HRQL was measured by the Mandarin version of the 36-item Short Form. We used the validated Mandarin version of the Self-Efficacy for Managing Chronic Disease 6-Item Scale to assess patients’ self-management efficacy. The validated three-item Brief Health Literacy Screening (BHLS) was used to measure the patients’ health literacy.

A structural equation model was constructed, and *p* ≤ 0.05 was taken as significant.

**Results:**

Demographic characteristics, health literacy and self-management efficacy have all significant effects on HRQL. Age, education level, self-management efficacy and health literacy were significantly related to the HRQL. The constructed model had a good fit for the data according to the model fit indices. Based on the model, health literacy (*r* = 0.604, *p* = 0.029) and Self-management efficacy (*r* = 0.714, *p* = 0.018) have a significant impact on HRQL. Demographic characteristics were inversely related to HRQL (*r* = −0.419, *p* = 0.007), but have a significant impact on health literacy (*r* = 0.675, *p* = 0.029) and self-management efficacy (*r* = 0.379, *p* = 0.029). At the same time, self-management efficacy was positively correlated to health literacy (*r* = 0.413, *p* < 0.01).

**Conclusions:**

Age, education level, self-management efficacy and health literacy were all related to the HRQL of patient with hypertension, which means that patients who are more elderly and have lower education level, low self-management efficacy and poor health literacy get worse HRQL. This may imply the necessary to introduce routine assessment of health literacy and self-management efficacy into assessment procedures for hypertensive patients’ health management. Such assessment can help professionals to identify the population at greatest risk for poor health outcomes and low well-being in the future. In clinical practice, effective interventions such as direct guidance and education to raise the self-management efficacy and enhance health literacy might improve the HRQL of patients with hypertension.

**Trial registration:**

Retrospectively registered Chinese Clinical Trial Registry (ChiCTR-OOR-14005563).

Name of registry: Effects of the integrated delivery system and payment system of community-based intervention on rural patients of chronic diseases in Qianjiang District, China

Date of registration: Retrospectively registered 23 November 2014.

Date of enrolment of the first participant to the trial: 5 July 2012

## Background

Hypertension is a common disease of the cardiovascular system that causes approximately 71 million deaths worldwide each year; the mortality rate was 26.4% in 2000, and is expected to rise to 29.2% by 2025 [1]. Results of the fourth national health services survey in China have shown that great changes have taken place in the structure of 2-week sickness prevalence rates, with chronic diseases making up a growing proportion of this rate [2]. As a result, chronic diseases have become the leading cause of ill health in China. Among chronic diseases, hypertension affects the most patients and the rate of increase of patients with hypertension is the most rapid. This growth of patients with hypertension in China is occurring at a higher speed in rural areas than urban areas [2]. Furthermore, hypertensive patients in rural areas have the characteristics of low health literacy and poor self-management ability, as hypertension has been effectively controlled in low percentage of these patients [2]. The complex nature of disease and the feeling of being ill will diminish quality of life and result in decreased satisfaction with daily life [3].

Health-related quality of life (HRQL), which refers to perceived physical and mental health and function, is an important health indicator in medical interventions and health surveys [4–6]. It is an assessment of how the individual’s well-being may be affected over time by a disease, disability, or disorder.

The current concept of health-related quality of life acknowledges that subjects put their actual situation in relation to their personal expectation. The latter can vary over time, and react to external influences such as length and severity of illness, family support, etc. As with any situation involving multiple perspectives, patients’ and physicians’ rating of the same objective situation have been found to differ significantly. Consequently, health-related quality of life is now usually assessed using patient questionnaires. These are often multidimensional and cover physical, social, emotional, cognitive, work- or role-related, and possibly spiritual aspects as well as a wide variety of disease related symptoms, therapy induced side effects, and even the financial impact of medical conditions (https://en.wikipedia.org/wiki/Quality_of_life_(healthcare)).

Additionally, health-related quality of life research may be used as the final step in clinical trials of experimental therapies (https://en.wikipedia.org/wiki/Quality_of_life_(healthcare)).

HRQL of hypertensive patients is not only affected by the disease itself but also by some subjective factors such as health literacy, self-management and psychological factors [7]. The interaction of the various factors increases the complexity and the difficulty of research. Although numerous studies [8–12] have investigated the objective factors, few studies have evaluated the subjective factors and mechanism of interaction. Saleem et al. [13] evaluated the association between HRQL and disease state knowledge among hypertensive patients in Pakistan. The results of this study suggested that hypertension knowledge was weakly associated with HRQL. Jayasinghe et al. [14] explored the HRQL of a large number of hypertensive patients in Australia. The study results indicated that patients with different gender and age showed different physical and/or mental characteristics. Vathesatogkit et al. [15] examined and compared the effects of different health states on HRQL in a Thai population. The researchers found that awareness of diabetes and hypertension negatively influenced the mental component summary (MCS) but not influence the physical component summary (PCS). The study results indicated that sex and age were related to HRQL. However, most of the research conducted has focused on the influence of objective factors and ignored the role of subjective factors [16, 17]. In addition, relevant research of the pathway of how these factors affect HRQL is lacking.

Health literacy is the ability to obtain, read, understand and use healthcare information to make appropriate health decisions and follow instructions for treatment (https://en.wikipedia.org/wiki/Health_literacy). Health Literacy has been defined as the cognitive and social skills which determine the motivation and ability of individuals to gain access to, understand and use information in ways which promote and maintain good health. Health Literacy means more than being able to read pamphlets and successfully make appointments. By improving people’s access to health information and their capacity to use it effectively, health literacy is critical to empowerment (https://en.wikipedia.org/wiki/Health_literacy).

Many factors determine the health literacy level of health education materials or other health interventions: reading level, numeracy level, current state of health, language barriers, cultural appropriateness, format and style, sentence structure, use of illustrations, interactiveness of intervention, and numerous other factors will affect how easily health information is understood and followed. So Health literacy is one of the important factors that influence patients’ HRQL and whether they could obtain the health services they need.

Self-management efficacy focus on the confidence in the ability to conduct self- management activities. At present, self-management efficacy has been widely concerned in the management of chronic diseases. It is an important factor that influence the self-management behavior of patients, which can improve the self-management ability by improving the self-management efficacy of patients [18].

The purpose of this study was to evaluate the association between HRQL and health literacy and self-management efficacy and how the factors affect the HRQL among hypertensive patients in rural western China. A structural equation model was constructed to explore how these factors predict the HRQL of rural patients with hypertension and suggestions are provided for interventions and strategies to improve the HRQL of these patients.

In view of this, HRQL of hypertensive patients in less developed areas is more worthy of attention, we have chosen a typical representative district of the less developed region of China——Qianjiang District, Chongqing, as a sample area.

Qianjiang District is a typical rural area locates in southeast Chongqing, the only municipality who sits in the deeper part of southwest China and thus is less developed compared to the other three municipalities. Qianjiang had a population size of 550,000 people whose average income per capita per year in the past 5 years was under US$480, relatively half of the whole nation’s spectrum (data resource: Qianjiang District National Economic and Social Development Statistics Bulletin 2001–2012). Qianjiang has in total 30 communities and 24 of them are rural ones. The average town population size is about 12,000, and every town has around 10 villages. Rural people accounted for 80% of the total population. Over 90% of the rural people have enrolled in the New Rural Cooperative Medical Scheme, which allow them to get nearly 60%(2012) reimbursement of the total in-patient medical expenditure from the medical insurance fund.

## Methods

### Study population and design

This was a cross-sectional study of baseline data from a clustered randomized controlled trial. The study design had passed a cross-national peer review and accepted grants by the China Medical Board. It was also registered in the Chinese Clinical Trial Registry (ChiCTR-OOR-14005563).

Six towns: Apengjiang, Zhuoshui, Shihui, Jinxi, Fengjia and Shijia towns were selected randomly from the 24 rural communities with a combined consideration of population size, social development and geographic position.

A cross-sectional study based on questionnaire responses was conducted to explore HRQL among hypertensive patients. The prevalence of hypertension was 17.7% in the nearest epidemiological investigation before trial (Qianjiang Animal Disease Surveillance and Epidemiological Investigation, 2012); therefore, there were theoretically nearly 2,470 hypertensive patients in average in each town.

Six thousand eight hundred thirty-three hypertensive patients in total were registered as a managed chronic patient in the database of the new rural cooperative medical scheme according to the baseline survey in 2012. Participants were selected from the database according to the following standards from the sample towns.

The inclusion criteria of participants for investigation and following-up were: (1) patients who had been registered as a managed chronic patient between year 2008 to January 2012, which meant the participants were all aged over 35 and had an official health records including their basic demographic information, symptoms and risk factors, and with a history of hypertension no less than 6 months and taking BP records at least four times a year; (2) patients who had been consistently enrolled in the new rural cooperative medical scheme; (3) patients who constantly reside in his/her own cluster, which was defined for at least 1 year before intervention and at least 6 months after and must ensure that have a complete BP record.

The exclusion criteria were: (1) those who had a stable BP history (consistently under 120/80 mm Hg) longer than 1 year and thus would not admit having chronic diseases or refuse to take medicines; (2) those who were estimated for a life expectancy less than 2 years due to old age, venerable situations or severe complications such as cerebral infarction or pancreatic cancer, thus would potentially call for apparent extra samples; (3) those who would probably get lost in follow-ups with high chances to go out, which was recognized for at least 6 months away yearly during the intervention period for reasons as migration for work, education or kinship-care-seeking; (4) those who would hardly visit or investigate due to intellectual or activity incompetence; (5) those who mentally damaged or communication incapable; (6) those who refuse to cooperate due to personal reasons.

According to the standards, a total of 1000 patients which accounted for 32.4% of the total were selected from the six towns at random.

In order to ensure sufficient statistical power, we took three steps to decide the sample size: (1) Screened in accordance with the criteria in the registered patients in the database for access to the samples; (2) Calculated the sample size that meet the requirement of the most difficult quota; (3) Estimate the statistical power of other quotas in this sample size to check whether it can meet all the requirements. We used PASS(11.0) to estimate the statistical power. α < 0.05 and statistical power >80% were taken as significant, 882 samples could fully meet all the requirements.

### Ethical approval and questionnaire

The survey was approved by the Huazhong University of Science and Technology Ethics Committee. Each patient who participated in the study was informed of the nature and objectives of the survey. A consent form was signed before data collection from each patient.

A standardized questionnaire adapted from a previous validated WHO questionnaire was used for the survey which included detailed questions about patient’s socio-demographic characteristics and self-reported information.

Patients’ HRQL was measured by the Mandarin version of the 36-item Short Form. We used the validated Mandarin version of the Self-Efficacy for Managing Chronic Disease 6-Item Scale to assess patients’ self-management efficacy. The validated three-item Brief Health Literacy Screening (BHLS) was used to measure the patients’ health literacy.

The HRQL of hypertensive patients was measured with the Chinese version of the 36-item Short Form (SF-36), which has been widely used [19] to evaluate patient health. The SF-36 consists of eight dimensions: physical function (PF), role limitations due to physical problems (RP), bodily pain (BP), general health (GH), vitality (VT), social function (SF), role limitations due to emotional problems (RE), and mental health (MH). It can also be divided into two summaries: PCS (PF, RP, BP and GH) and MCS (RE, SF, MH and VT). After administration of the questionnaire, all domain scores are converted with the following formula for comparison: SS = (Rs − Min) × 100/R, where SS, Rs, Min and R represent standardized score, raw score, minimum score of the dimension and range of scores in the dimension, respectively, so HRQL variable, an endogenous latent variable is a continuous variable . The higher the SF-36 score gets, the better the level of HRQL is.

Self-Efficacy for Managing Chronic Disease 6-Item Scale was developed by the Patient Education Research Center of Stanford University of the United States in 1980s, which was widely used all over the world for the evaluation of the effect of the self-management of patients with chronic disease.

Six items were included in the Self-management efficacy Subscale to help to assess that how confident patients are in doing certain activities. For each of the questions, patients choose the score that corresponds to the confidence that they can do the tasks regularly at the present time, with the following chart as an example.

**Table:** 

1. How confident are you that you can keep the fatigue caused by your disease from interfering with the things you want to do?											
Not at all confident	1	2	3	4	5	6	7	8	9	10	Totally confident

Items were scored on a 10-point Likert scales, with a higher score indicating better self-manage efficacy.

Hypertensive patients’ health literacy was measured by the validated three-item Brief Health Literacy Screening (BHLS) [20] which has been used in earlier studies on health literacy [21, 22]. The items of the BHLS are the following:

1. How often do you have someone help you read hospital materials?
2. How confident are you filling out medical forms by yourself?
3. How often do you have problems learning about your medical condition because of difficulty understanding written information?

These items were answered on a 5-point Likert scale (1–5). By reversing the scores on the second question and then summing up the scores of all three questions, a continuous total score (3–15) was calculated, with higher scores indicating higher levels of health literacy [20].

After the raw data were standardized, a structural equation model was constructed, and *p* ≤ 0.05 was taken as significant.

### Data collection

Supervision was provided by graduates from the School of Medicine and Health Management of Tongji Medical College of Huazhong University of Science and Technology. All supervisors were fully trained for 2 weeks before surveying and had completed related professional courses in medicine and health management. To ensure the quality of the survey, all questionnaires were checked and signed by another supervisor.

### Data management and analysis

The database was established using EpiData Version 3.1 (The EpiData Association, Odense, Denmark), and all questionnaires were coded and double-entered by two independent professional data-entry staff. Data were analyzed using SPSS 18.0 software (SPSS Inc., Chicago, IL, USA). Continuous variables were presented as mean ± standard deviation and categorical data were shown as frequencies and percentages. Mann–Whitney *U* and Kruskal–Wallis tests were used to test the significance among variables. *P* values less than 0.05 were taken as significant. IBM SPSS AMOS 20.0 (IBM Corp., Chicago, IL, USA) was used to establish structural equation modeling (SEM). HRQL, demographic characteristics, health literacy and self-management efficacy were set as latent variables, and the corresponding entries were set as observed variables. The model was constantly refined and re-estimated to verify model fit and to select the best-suited model.

## Results

### Demographic characteristics of the sample

A total of 1000 patients were enrolled in the survey, of which 986 completed questionnaires, and 882 questionnaires were in full compliance with the requirements. The response rate was 98.6%.

Among the 882 respondents, 387 (43.9%) were men and 495 (56.1%) were women. Respondent ages ranged from 35 to 92 years, with a mean age of 65.87 years. The majority (80%) had a primary school level of education or lower. The average yearly family income and yearly family medical costs were 17618.64 yuan and 3405.61 yuan, respectively. The specific demographic data of the study are presented in Table 1.

**Table 1:** Characteristics of the study population (*n* = 882)

Variables	Frequency(n)	Percent
Gender		
Male	387	43.9
Female	495	56.1
Age (Mean ± SD) = 65.87 ± 10.73		
35–46	53	6.0
47–59	186	21.1
60–72	413	46.8
≥ 73	230	26.1
Education		
Illiterate	303	34.4
Primary school	415	47.1
Secondary school	137	15.5
High school	10	1.1
University or higher	17	1.9
Family yearly income(RMB)		
< 5000	183	20.7
5000–10000	133	15.1
10000–20000	228	25.8
20000–30000	154	17.5
≥ 30000	184	20.9
Family yearly medical cost(RMB)		
< 600	177	20.1
600–1500	221	25.1
1500–3000	179	20.3
3000–1000	217	24.6
≥ 10000	88	10.0

### Self-management efficacy and health literacy of hypertensive patients

As Tables 2 and 3 show, 40.6% of people scored between 31 and 45 in self-management efficacy, and 56.3% of patients had scored between 12 and 15 in health literacy.

**Table 2:** Self-management efficiency of the study population (*n* = 882)

Self-management efficiency score	Frequency	Percent
≤15	55	6.2
16–30	215	24.4
31–45	358	40.6
45–60	254	28.8

**Table 3:** Health literacy of the study population (*n* = 882)

Health literacy score	Frequency	Percent
≤3	30	3.5
4–7	159	18.0
8–11	196	22.2
12–15	497	56.3

### SF-36 HRQL and influencing factors analysis

Table 4 shows the HRQL scores in all patients, and the relationship between demographic characteristics, health literacy and self-management efficacy and HRQL. With the results of Chew test, we could see with increasing age, HRQL scores decreased, and different age groups had significantly different MCS scores (*p* = 0.050), but the scores were not significantly different in PCS. There were also significant differences between education level groups. The HRQL score increased with education level; the *P* values for the differences between groups in PCS score were < 0.001, and MCS score was 0.001. The PCS score was significantly different for different levels of hypertension cognition (*p* = 0.029), and the MCS score was significantly different for different levels of health literacy (*p* = 0.001). Finally, sex, yearly family income and yearly family medical costs did not influence HRQL.

**Table 4:** HRQL scores in hypertensive patients (*n* = 882)

Variable	Frequency	Mean of PCS	SD of PCS	*P* value	Mean of MCS	SD of MCS	*P* value
Gender							
Male	387	34.85	20.32	0.146	50.15	23.43	0.157
Female	495	32.80	19.55		47.83	21.97	
Age							
35–46	51	36.20	18.15	0.530	54.78	22.98	0.050*
47–59	182	33.02	19.84		45.80	22.24	
60–72	401	33.84	20.37		49.14	22.59	
≥ 73	248	33.46	19.62		49.40	22.96	
Education							
Illiterate	303	29.94	18.03	0.000*	46.58	21.12	0.001*
Primary	415	33.93	20.21		48.30	23.82	
Secondary	137	39.66	20.98		52.63	21.66	
High school	10	39.92	16.63		60.42	17.35	
University	17	43.46	22.85		65.56	19.14	
Family yearly income (RMB)							
< 5000	183	31.45	19.28	0.575	46.38	22.80	0.501
5000–10000	132	35.71	21.83		49.24	24.58	
10000–20000	230	33.86	18.88		49.47	21.88	
20000–30000	153	34.40	20.42		50.08	22.87	
≥ 30000	184	33.71	19.92		49.23	21.83	
Family yearly medical cost (RMB)							
< 600	177	32.60	20.19	0.684	46.93	24.22	0.738
600–1500	221	34.56	19.35		49.67	21.65	
1500–3000	179	33.24	20.04		48.68	22.92	
3000–1000	217	33.93	21.09		48.86	22.59	
≥ 10000	88	34.13	17.66		50.97	21.52	
Self-management efficacy score							
≤ 15	55	37.93	23.06	0.029*	49.28	22.17	0.084
16–30	215	36.30	20.47		52.06	22.79	
31–45	358	31.61	18.63		47.69	23.03	
46–60	254	33.53	20.17		47.67	21.91	
Health literacy score							
≤ 3	31	35.51	25.36	0.131	51.28	24.03	0.001*
4–7	159	31.82	20.86		43.62	23.42	
8–11	196	31.97	18.76		45.99	21.75	
12–15	496	34.87	19.63		51.50	22.29	

Physical Component Summary (PCS): The mean of physical function (PF); role limitations due to physical problems (RP); bodily pain (BP); general health (GH)

Menal Component Summary (MCS): The mean of vitality (VT); social function (SF); role limitations due to emotional problems (RE); mental health (MH)

*P < 0.05

### Results of SEM and construct validity

In this study, based on the literature review [7–18] and the results above, demographic characteristics, health literacy and self-management efficacy are all associated with HRQL,we proposed the initial structural equation model.

**Fig. 1:**
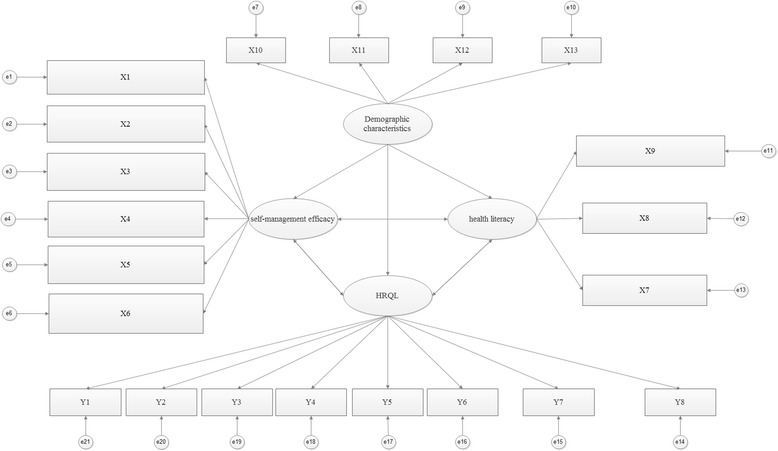
Full model path analysis diagram. Y1: Pysical function (PF). Y2: General health (GH). Y3: Vitality (VT). Y4: Role limitations due to physical problems (RP). Y5: Role limitations due to emotional problems (RE). Y6: Social function (SF). Y7: Bodily pain (BP). Y8: Mental health (MH). X1: How confident are you that you can keep the fatigue caused by hypertension from interfering with the things you want to do? *X*2: How confident are you that you can keep the physical discomfort or pain of hypertension from interfering with the things you want to do? X3: How confident are you that you can keep the emotional distress caused by hypertension from interfering with the things you want to do? X4: How confident are you that you can keep any other symptoms or health problems you have from interfering with the things you want to do? X5: How confident are you that you can do the different tasks and activities needed to manage your health condition so as to reduce you need to see a doctor? X6: How confident are you that you can do things other than just taking medication to reduce how much you illness affects your everyday life? X7: How often do you have someone help you read hospital materials? X8: How confident are you filling out medical forms by yourself? X9: How often do you have problems learning about your medical condition because of difficulty understanding written information? X10: Age. X11: Education level. X12: Yearly family income. X13: Yearly family medical cost

According to the research hypothesis, the path analysis diagram of the whole model is established as Fig. 1.

We conducted identification test of the structural equation model according to the principle as following: The free parameters of the model can not be more than the total variance and covariance of the observed variables. For example, there are n + m observable variables in total in the model, and we take *t* as the number of parameters in the model. A necessary condition for the identification of the model is that *t* ≤ (m + n)(m + n + 1)/2. In our model, we have 64 parameters in total, and obviously 58 < (8 + 13)(8 + 13 + 1)/2, so the model could be identified.

The maximum likelihood ratio was used as the method of estimation, and the model fit index was used to check the fitting degree of the theoretical model to the data. AMOS provides a variety of model fit indices, for example, absolute fit index: chi-square (*χ* ^2^), degrees of freedom (df), goodness-of-fit index (GFI), root mean square residual (RMR), standardized root mean square residual (SRMR) and root mean square error of approximation (RMSEA); relative fit index: normed fit index (NFI), Tucker-Lewis index (TLI), comparative fit index (CFI) and incremental fit index (IFI); and information index: Akaike’s information criterion (AIC) and consistent Akaike’s information criterion (CAIC). The results of model fitness are shown in Table 5. The model showed a good fit of the data based on these criteria.

**Table 5:** Model fit index

Variable	*χ* ^2^(df)	GFI	RMSEA	NFI	TLI	CFI	IFI	AIC
Fit index	298.3(150)	0.967	0.032	0.937	0.961	0.969	0.969	408.3
Reference value	-	>0.9	<0.05	>0.9	>0.9	>0.9	>0.9	>400

All the latent variables, the corresponding observed variables, and the results of the regression weight significance test are shown in Table 6. The results showed that there were strong correlations between observed variables and their corresponding latent variables (most regression weights were higher than 0.5); these associations were consistent with the theoretical constructs.

**Table 6:** The latent variable, observed variable, and the result of regression weight significant test

Latent variable	Observed variable	Regression weights	SE	CR	*P*-value	Stantard regression weights
Demographic characteristics	Age	1.000				0.438
	Education	−1.107	.281	−3.940	**	−.503
	Family yearly income	−2.438	.623	−3.911	**	−.725
	Family yearly medical cost	−1.508	.462	−3.262	.001	−.620
Self-management efficacy	How confident are you that you can keep the fatigue caused by hypertension from interfering with the things you want to do?	1.000				.354
	How confident are you that you can keep the physical discomfort or pain of hypertension from interfering with the things you want to do?	1.770	.208	8.545	**	.675
	How confident are you that you can keep the emotional distress caused by hypertension from interfering with the things you want to do?	1.121	.186	6.026	**	.303
	How confident are you that you can keep any other symptoms or health problems you have from interfering with the things you want to do?	2.248	.262	8.579	**	.734
	How confident are you that you can do the different tasks and activities needed to manage your health condition so as to reduce you need to see a doctor?	1.469	.180	8.145	**	.580
	How confident are you that you can do things other than just taking medication to reduce how much you illness affects your everyday life?	−1.317	.183	−7.214	**	-.503
Health literacy	How often do you have someone help you read hospital materials?	1.000				.439
	How confident are you filling out medical forms by yourself?	1.770	.134	13.227	**	.881
	How often do you have problems learning about your medical condition because of difficulty understanding written information?	1.922	.149	12.882	**	.937
	Pysical function (PF)	2.132	.243	9.124	**	.684
Health-related quality of life	General health (GH)	1.000				.452
	Vitality(VT)	.845	.098	8.665	**	.321
	Role limitations due to physical problems (RP)	2.333	.258	9.053	**	.614
	Role limitations due to emotional problems (RE)	2.554	.261	9.787	**	.569
	Social function (SF)	2.067	.202	10.223	**	.699
	Bodily pain (BP)	1.535	.156	9.824	**	.679
	Mental health (MH)	1.831	.174	10.505	**	.602

**P < 0.01

### The final model and interpretation

**Fig. 2:**
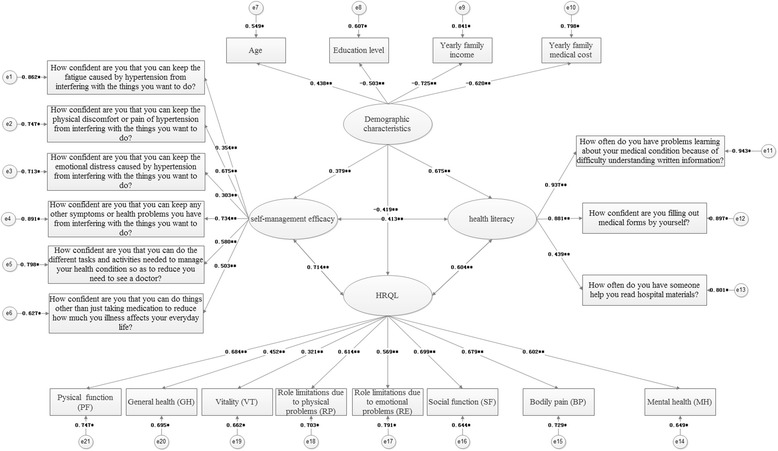
The structure of HRQL presented by structural equation modeling analysis (Chi - Square = 298.3, df = 150, *P* - value = 0.000, RMSEA = 0.032). Note: *p<0.05; **p<0.01

Figure 2 shows the pathways of the final model and their regression weight indices. Health literacy was significantly related to HRQL (*r* = 0.604, *p* = 0.029), and demographic characteristics were inversely related to HRQL (*r* = −0.419, *p* = 0.007). Self-management efficacy has a significance impact on HRQL (*r* = 0.714, *p* < 0.01) and at the same time was positively related to health literacy (*r* = 0.413, *p* < 0.01). Besides, demographic characteristics have significant impact on health literacy (*r* = 0.675, *p* < 0.01) and self-management efficacy (*r* = 0.379, *p* < 0.01).

## Discussion

To the best of our knowledge, this study is the first to explore how health literacy and self-management efficacy affect the HRQL in patients with hypertension in a rural area of southwestern China.

Hypertension is a common and frequently occurring disease of the cardiovascular system. Patients with hypertension that is poorly controlled are at an increased risk of developing cardiovascular and cerebrovascular diseases, and are at an increased risk of mortality from these diseases [23]. Hypertension has also become an important public health problem in China. However, to control the disease, it is not enough to focus on the disease itself and its treatment, in reality, the subjective factors such as health literacy and self-management efficacy also have great impact to the HRQL. To a certain extent, paying more attention to subjective factors is conducive to the prevention and control of hypertension. Therefore, in this study, we explored the influence of health literacy and self-management efficacy on HRQL in a rural population of southwestern China with hypertension, and analyzed how they affect the HRQL.

Our findings indicate that patients who had higher health literacy and self-management efficacy get better HRQL. However patients who are more elderly with lower education level get worse HRQL.

This study demonstrates that HRQL had a significant relationship with age, education level, health literacy and self-management efficacy. The findings are not completely consistent with those of other studies. Saleem et al. [13] explored the HRQL profile of a hypertensive population in Pakistan. The study found that education, income and locality had a significant relationship with HRQL, but there was no significant difference between different age groups. Wang et al. [24] found that hypertension markedly impaired quality of life, and comorbidity further deteriorated HRQL among people with hypertension in China. Zyoud et al. [12] assessed adherence and HRQL in hypertensive patients in the Middle East; the results showed that patients with a high adherence had the highest HRQL. In this study, similar to patients of other regions and cultures, age and education level had a significant relationship with HRQL in terms of both physical and mental health. Our study did not find that yearly family income and yearly family medical costs influenced HRQL, which is not consistent with other studies [25]. One possible explanation for these results is that all the hypertensive patients who participated in the program are in rural cooperative medical care, therefore, cost is not a big barrier for seeking medical advice.

We found that self-management efficacy influenced the PCS, but had no effect on the MCS. By contrast, health literacy influenced the MCS, but had no effect on PCS. These findings are in accordance with those of other studies [13, 15]. However, some studies have shown that increased awareness of hypertension is related to lower HRQL [12, 26]. Other studies [12, 20–22, 27–30] also reported that patients with a high adherence had the highest HRQL. In the process of investigation, we found that at early stages of the disease when patients had mild symptoms they often did not take the initiative to learn about hypertension and did not accept medical treatment regularly. However, when obvious symptoms appeared, patients would learn about hypertension and accept expensive medical costs to treat comorbidities of hypertension, at a point when their HRQL had been irreversibly impaired. That indicates low health literacy is prevalent in the rural areas of southwestern China, and patients had not realized the importance of self-management. This observation was not in accordance with the study by Zyoud et al. [12], which found that participants with low HRQL were more likely to have lower adherence to anti-hypertensive medications. One explanation for these results might be the differences in health concepts and cultures of different regions and nations. However, this discrepancy has significant implications for the secondary prevention of hypertension. In the early stage of the disease, through self-management education, and through direct guidance and management of the disease, improvement of HRQL is still possible. In the advanced stages of disease, even if patients have a high self-management efficacy and cooperate actively with treatment, improvement of HRQL is limited.

All indices suggest that the final structural equation model reasonably fits the data, and it is consistent with the theoretical constructs. The model revealed that health literacy was positively associated with HRQL. Hence, an increase in the health literacy predicted higher HRQL. Self-management efficacy is positive related to health literacy, and is indirectly related to HRQL. These findings lend further support to our viewpoint that improvement of the self-management predicts higher patient HRQL. Based on a structural equation model, we see that the greater the age of patients and the lower their education level, the lower is their HRQL.

There are limitations in our study. First, this study is cross-sectional, therefore, the causal nature of the influencing factors cannot be determined. To further investigate the issue, a prospective design should be used. Second, the data that were derived from different data sources may lead to more generalized results at which we were not good enough. Third, because some of the data information is not detailed enough, we could not stratify the samples to do further analysis which may give a better explanation for the association between health literacy,self-management efficacy and HRQL.

## Conclusions

Professionals working with hypertensive patients should be aware of the association between HRQL and health literacy and self-management efficacy in health management. This may also imply the necessary to introduce routine assessment of health literacy and self-management efficacy into assessment procedures for hypertensive patients. Such assessment can help professionals to identify the population at greatest risk for poor health outcomes and low well-being in the future. If low health literacy and self-management efficacy cause a decrease in HRQL among hypertensive patients, future interventions should focus on mitigating the negative effects of them.

According to the characteristics of the hypertension itself, especially in the early stage of the disease, prevention before disease attack and health management is more effective than medical treatment. And good health literacy is absolutely a necessary condition of patients’ initiative prevention, as it can help people to realize how this disease attack and develop more healthy habits so as to help reduce the incidence or delay disease progression. Meanwhile, enhancing self-management efficacy can help patients to get healthy lifestyle, which would help avoiding hypertension deteriorating. Thus, the HRQL of patients with hypertension might be improved by encouraging patients to carry out better self-management and enhance health literacy, especially in the early stage of the disease. In clinical practice, with some kind of educational materials and effective interventions such as direct guidance for patients with low health literacy and poor self-management efficacy, we can improve health management in the society and mitigate the adverse health effects of low health literacy and poor self-management efficacy so as to help hypertensive patients to get better HRQL.

## Abbreviations

*χ*^2^: Absolute fit index: chi-square
AIC: Information index: Akaike’s InformationCriterion
BHLS: Brief Health Literacy Screening
BP: Bodily pain
CAIC: Consistent Akaike’s Information Criterion
CFI: Comparative Fit Index
Df: Degrees of freedom
GFI: Goodness-of-Fit Index
GH: General health
HL: Health literacy
HRQL: Health-related quality of life
IFI: Incremental Fit Index
MCS: Mental component summary
MH: Mental health
NFI: Relative fit index: Normed Fit Index
PCS: Physical component summary
PF: Physical function
RE: Role limitations due to emotional problems
RMR: Root Mean Square Residual
RMSEA: Root Mean Square Error of Approximation
RP: Role limitations due to physical problems
SEM: Structural equation modeling
SF: Social function
SF-36: 36-item Short Form
SME: Self-management efficacy
SRMR: Standardized Root Mean Square Residual
TLI: Tucker-Lewis Index
VT: Vitality
WHO: World Health Organization
